# Ran Binding Protein 9 (RanBP9) is a novel mediator of cellular DNA damage response in lung cancer cells

**DOI:** 10.18632/oncotarget.7813

**Published:** 2016-03-01

**Authors:** Dario Palmieri, Mario Scarpa, Anna Tessari, Rexhep Uka, Foued Amari, Cindy Lee, Timothy Richmond, Claudia Foray, Tyler Sheetz, Ashley Braddom, Christin E. Burd, Jeffrey D. Parvin, Thomas Ludwig, Carlo M. Croce, Vincenzo Coppola

**Affiliations:** ^1^ Department of Molecular Virology, Immunology and Medical Genetics, College of Medicine, 43210 Columbus, OH, USA; ^2^ Solid Tumor Biology Program, Comprehensive Cancer Center, The Ohio State University, 43210 Columbus, OH, USA

**Keywords:** RanBP9, RanBPM, ATM, DNA damage, ionizing radiation

## Abstract

Ran Binding Protein 9 (RanBP9, also known as RanBPM) is an evolutionary conserved scaffold protein present both in the nucleus and the cytoplasm of cells whose biological functions remain elusive.

We show that active ATM phosphorylates RanBP9 on at least two different residues (S181 and S603). In response to IR, RanBP9 rapidly accumulates into the nucleus of lung cancer cells, but this nuclear accumulation is prevented by ATM inhibition. RanBP9 stable silencing in three different lung cancer cell lines significantly affects the DNA Damage Response (DDR), resulting in delayed activation of key components of the cellular response to IR such as ATM itself, Chk2, γH2AX, and p53. Accordingly, abrogation of RanBP9 expression reduces homologous recombination-dependent DNA repair efficiency, causing an abnormal activation of IR-induced senescence and apoptosis.

In summary, here we report that RanBP9 is a novel mediator of the cellular DDR, whose accumulation into the nucleus upon IR is dependent on ATM kinase activity. RanBP9 absence hampers the molecular mechanisms leading to efficient repair of damaged DNA, resulting in enhanced sensitivity to genotoxic stress. These findings suggest that targeting RanBP9 might enhance lung cancer cell sensitivity to genotoxic anti-neoplastic treatment.

## INTRODUCTION

Several cancer treatments, and lung cancer in particular, cause DNA damage and activation of the cellular response to DNA damage (DNA-Damage Response, DDR) [1, 2]. The DDR protects the genome from exogenous and endogenous insults, maintains cellular homeostasis, and prevents premature aging and cancer [3–7]. Upon Ionizing Radiation (IR) or genotoxic chemotherapies, the most deleterious type of DNA damage is represented by double strand breaks (DSBs) that are repaired by either Homologous Recombination (HR) or Non-Homologous End Joining (NHEJ) [1–3]. Taking advantage of the recombination between sister chromatids, HR allows an error-free repair of DNA damage [3]. Following DNA DSBs, sensor proteins are recruited to the damage sites forming large nuclear structures (damage foci). In turn, sensors of DNA damage mediate the activation of transducers, powerful protein kinases that trigger the activation of effector proteins. Effectors determine activation of cell-cycle checkpoints, chromatin remodeling, and the resolution of the DNA damage. However, if the damage is substantial or beyond repair, they trigger senescence and apoptosis to prevent the spread of genetically altered cells [8].

ATM, the protein encoded by the gene mutated in Ataxia-Telangiectasia, is the primary transducer of the response to DNA-DSB, phosphorylating hundreds of target proteins involved in the DDR [8–10]. Following DSBs induction, activation of ATM is dependent on the recruitment at the damage site of the MRE11-RAD50-NBS1 (MRN) complex, and is associated to several post-translational modifications of ATM itself, including auto-phosphorylation and acetylation [8, 10–17]. ATM phosphorylation is not considered anymore the primary mechanism of ATM activation, since this post-translational modification is dispensable for ATM function [18–20]. Conversely, ATM acetylation by KAT5 (Lysine Acetyl Transferase 5, aka Tip60) is essential for ATM activation following DNA damage [21]. Although intensively studied, the DDR is a complex signal transduction process and a complete list of all the players and mechanisms involved is still missing.

Human tumors display altered DNA repair ability, which can result in vulnerability potentially exploitable for therapeutic purposes [22–24]. For this reason, several groups are investigating the use of DNA-repair inhibitors (including ATM inhibitors [25]) to potentiate the efficacy of IR and genotoxic agents [26–28]. Further, factors involved in the DDR are used as biomarkers to predict responses to chemo- and radiotherapy [23, 29].

We investigated the role in the DDR of Ran Binding Protein 9 (RanBP9, also known as RanBPM), a protein whose biological functions are still unknown [30–34]. RanBP9 interacts with several factors involved in various cellular processes such as signal transduction, gene expression, cell adhesion and migration [35–37]. RanBP9-deficient mice show early perinatal lethality and sterility [38, 39]. RanBP9 has also been identified as the human homolog of the yeast Gid1 (glucose-induced degradation deficient 1) protein, member of a conserved multi-protein complex with E3 ubiquitin ligase activity responsible for the ubiquitination of fructose-1,6-bisphosphatase [40–42]. Relevant to the present work, RanBP9 becomes phosphorylated in cells exposed to different types of genotoxic stress including cisplatin, IR, UV exposure and osmotic shocks [43, 44]. Finally, a recent study dissected out the role of different regions of RanBP9 involved in its nuclear-cytoplasmic localization [34]. However, the stimuli and the mechanisms of RanBP9 nucleus-cytoplasmic shuttling remain largely unknown.

We report here the identification on RanBP9 of several putative target sites for transducers of the cellular DDR, including ATM. We demonstrate that ATM phosphorylates RanBP9 in response to IR and mediates its nuclear accumulation. We also show that RanBP9 participates in the full activation of ATM as well as of some of its targets and it is critically involved in HR-mediated DNA repair and in the cellular response to DNA damage.

## RESULTS

### RanBP9 is a novel target of ATM

**Figure 1:**
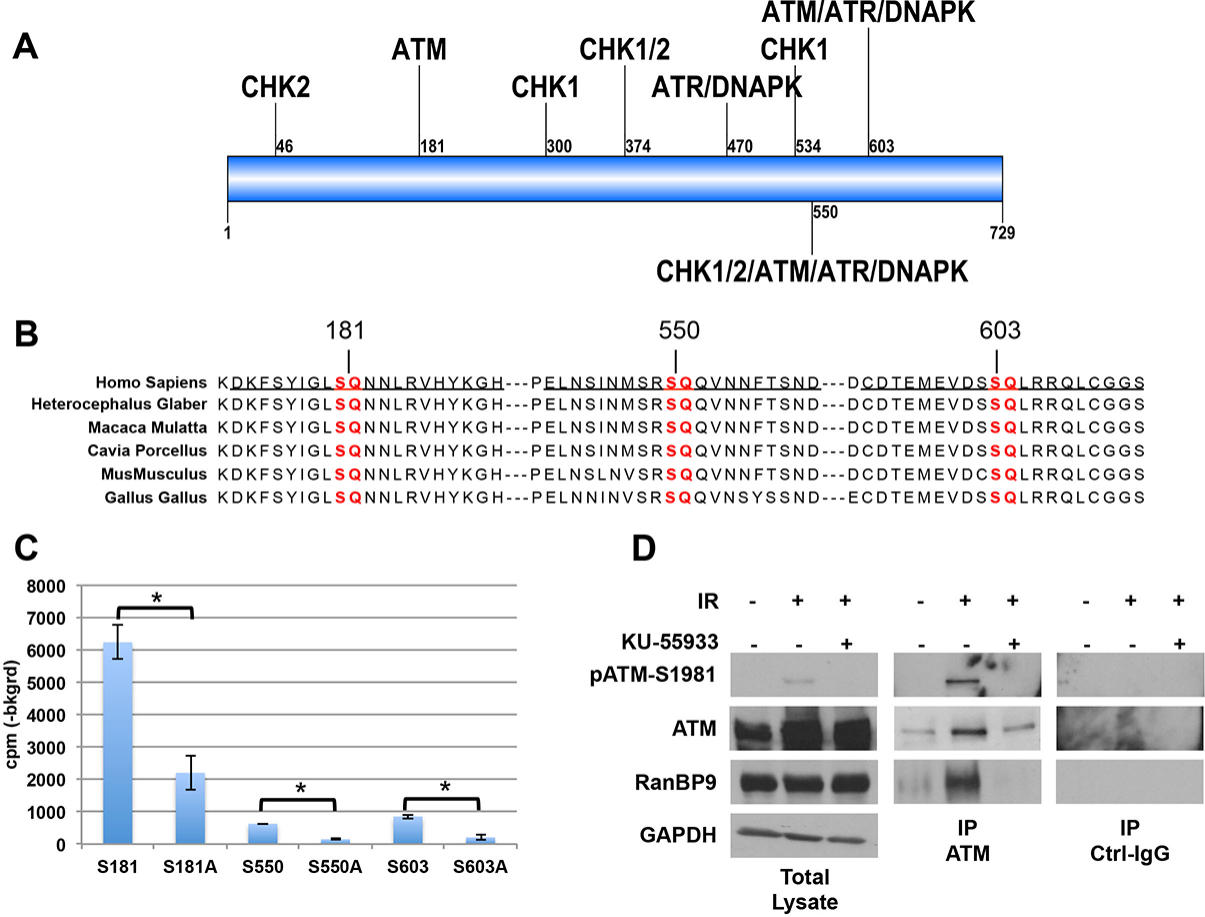
RanBP9 is a novel target of ATM (**A**) Schematic representation of RanBP9 protein showing putative phosphorylation sites for key kinases involved in the DNA-damage response (see also Supplementary Table 1). RanBP9 amino acid sequence (UniProt Q96S59) was analyzed using the Group-based Prediction System algorithm (GPS 2.0, http://bioinformatics.lcd-ustc.org/gps2) and searched for the presence of potential phosphorylation sites. The cut-off was set to a medium threshold with a false prediction rate below 6% for serine/threonine kinases. Supplementary Table 1 reports a comprehensive list of the predicted phosphorylation sites for ATM, ATR, DNA-PK, CHK1, and CHK2 with a prediction score higher than 2. Figure 1A shows the relative position of the putative phosphorylation sites with a score higher than 3. (**B**) Sequence conservation of potential ATM phosphorylation sites on RanBP9 protein. S/T-Q consensus sequences for ATM are highlighted in red. Peptides used in Figure 1C and Supplementary Figure 1B are underscored. (**C**) Radioactive kinase assay performed using recombinant active ATM kinase and peptides underlined in Figure 1B or their mutant S-A forms. A representative experiment (out of two independent experiments, performed in duplicate) + S.D. is reported. *p < 0.05. (**D**) Total cell lysates and immunoprecipitates (using either anti-ATM antibody or normal control IgG, Ctrl-IgG) were analyzed by WB to confirm that comparable levels of immunopurified ATM were used in kinase assay shown in Supplementary Figure 1B. Activation of ATM following IR treatment was evaluated using anti-pATM-S1981 antibody. RanBP9 antibody was used to confirm the interaction between ATM and RanBP9 in the indicated experimental conditions. GAPDH was used as loading control for total cell lysates.

To investigate the biological function of RanBP9, we performed a bioinformatic analysis of its protein sequence to identify potential phosphorylation sites (GPS 2.0, http://bioinformatics.lcd-ustc.org/gps2). This investigation identified several putative phosphorylation sites for the main kinases involved in cellular DDR (Figure 1A and Supplementary Table 1). However, in the present study, we only focused on sites potentially phosphorylated by ATM. Indeed, a previous large-scale screening for ATM substrates reported RanBP9 specific residue (S603) as *bona fide* target of ATM [45]. As shown in Figure 1B, the putative ATM phosphorylation sites on RanBP9 (S181, S550, and S603) are extremely conserved through evolution, supporting critical biological functions of these residues.

Based on these *in silico* predictions, we tested three different peptides, (underlined in Figure 1B) including the RanBP9 putative phosphorylation sites, as potential substrates for ATM kinase activity *in vitro* by kinase assay. Commercially available ATM active kinase was incubated with the indicated peptides or with their corresponding mutant versions where the predicted phosphorylated serine (S) was substituted by alanine (A). As show in Figure 1C, ATM was able to phosphorylate all the used wild-type peptides, but not their mutant S to A forms.

Then, we performed co-immunoprecipitation experiments using total cell extracts from lung cancer cell lines of different origin (A549, H460, and H1299), expressing detectable amounts of both ATM and RanBP9 proteins, plus or minus exposure to IR to activate the ATM kinase. Supplementary Figure 1A shows the co-immunoprecitipation between active-ATM (detected by anti-phosphoS1981) and RanBP9.

We then evaluated whether endogenous ATM purified from cell lysates phosphorylates RanBP9 on the predicted residues. To this end, we performed a non-radioactive kinase assays using immunopurified ATM from H460 cell extracts treated with 10 Gy of IR. As shown in Supplementary Figure 1B, significant ATM kinase activity was observed on S181 and S603 peptides. A modest but not significant phosphorylation was observed when S550 peptide was used.

Western blot (WB) analysis of total cell extracts and immunoprecipitates used in this assay confirmed that active ATM was only present in immunoprecipitates from IR-treated H460 cells (Figure 1D). The same analysis also revealed that RanBP9 co-immunoprecipitated with active ATM (Figure 1D), but not when ATM kinase activity was inhibited by the ATM-specific inhibitor KU-55933.

Taken together these data indicate that RanBP9 is a novel target of ATM and that ATM phosphorylates at least two different residues (S181 and S603) of RanBP9 following IR exposure.

### Nuclear accumulation of RanBP9 following IR depends on the activation of the ATM kinase activity

**Figure 2:**
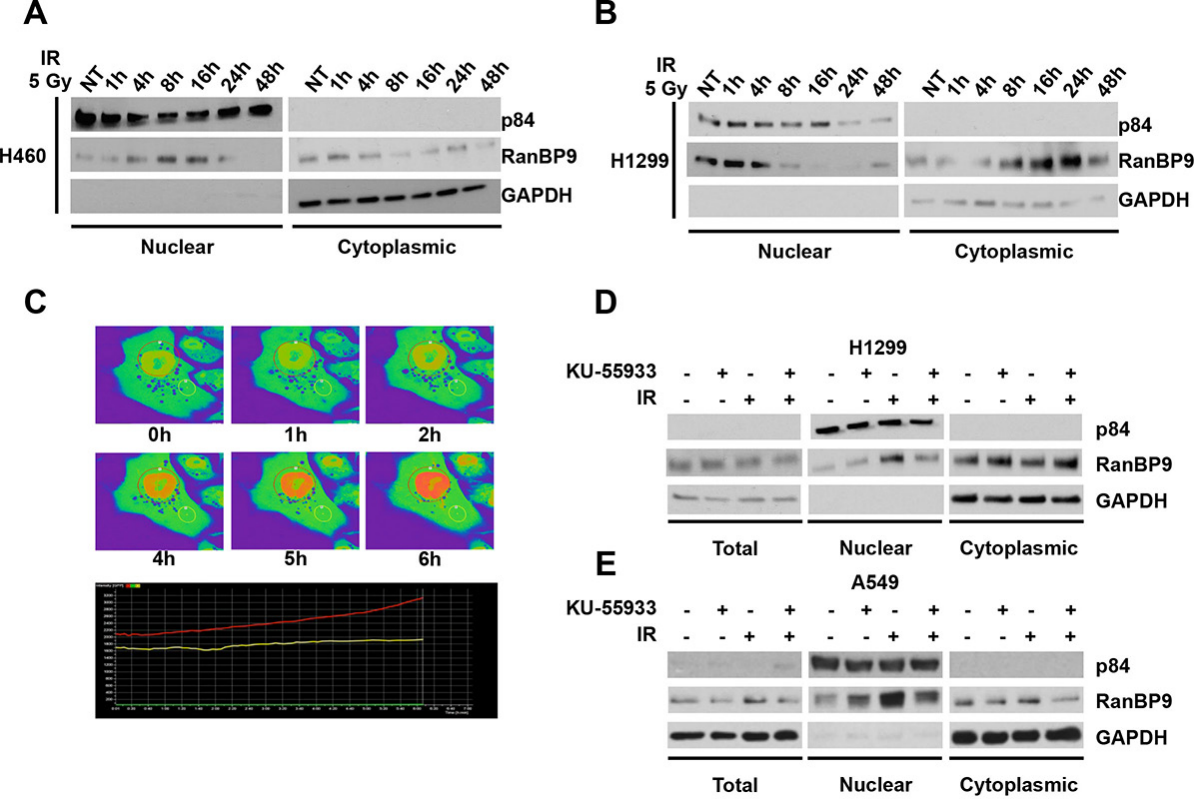
RanBP9 accumulates into the nucleus following IR exposure (**A**–**B**) Following IR exposure, H460 (A) and H1299 (B) lung cancer cells were harvested at the indicated time points and processed for nuclear/cytoplasmic protein extraction and fractionation. Nuclear and cytoplasmic fractions were analyzed through WB using the indicated antibodies. GAPDH and nuclear matrix protein p84 were used as cytoplasmic or nuclear loading controls, respectively. (**C**) H460 cells were transfected using a RanBP9-GFP expression vector. At 24 h from the transfection, cells were exposed to 10 Gy of IR and analyzed by live-imaging confocal microscopy for 6 h. Representative images for the indicated time points are shown. For quantitative analysis, images were processed using Nikon Element Viewer Software and regions of interest (ROI) are shown as red and yellow circles. Areas with higher levels of GFP-RanBP9 are indicated by orange/red pseudo-coloring to prevent saturation of the green signal. Graphical representation of cytoplasmic (yellow line) and nuclear (red line) RanBP9 levels over the time is also shown (lower panel). (**D**–**E**) Total cell extracts or nuclear/cytoplasmic protein fractions from H1299 (D) and A549 (E) lung cancer cells treated as indicated were analyzed by WB using the indicated antibodies. GAPDH and nuclear matrix protein p84 were used as total/cytoplasmic or nuclear loading controls, respectively. Where indicated, cells were left untreated (NT), pre-treated with 10 μM KU-55933 and/or treated with 5 Gy of Ionizing radiations (IR), and harvested 6 h after IR exposure.

Previous studies have indicated that RanBP9 is a protein able to move between the nucleus and the cytoplasm, but the molecular mechanisms regulating this shuttling are still unknown [43, 46, 47]. Interestingly, phosphorylation has been suggested as a potential post-translation modification regulating RanBP9 de-localization from the cytoplasm [44], and nuclear enrichment of RanBP9 following cisplatin treatment has been reported [43]. These earlier findings, along with our data demonstrating that ATM phosphorylates RanBP9, led us to investigate whether RanBP9 nuclear localization was dependent on ATM activation. To this aim, different lung cancer cell lines (H460, and H1299) were exposed to IR, harvested at different time points (0-48 h) and nuclear/cytoplasmic extracts were analyzed by WB. Figure 2A and 2B show that, in the analyzed cell lines, RanBP9 accumulated into the nucleus at short time points following IR exposure. Conversely, we observed increased RanBP9 cytoplasmic localization at longer time points (48 h), in agreement with previous reports from other groups [34]. Accordingly, live-imaging experiments using H460 cells expressing a RanBP9-GFP fusion protein also demonstrated that RanBP9 robustly accumulated into the nucleus at 4–6 h following IR (Figure 2C and Supplementary Video 1).

To test whether in our experimental conditions the nuclear accumulation of RanBP9 is dependent on ATM kinase activity, the indicated cell lines (Figure 2D–2E) were exposed to IR plus or minus KU-55933. Cell lysates were harvested at 6 h after IR and total, nuclear, and cytoplasmic extracts were analyzed by WB. In line with our previous results, IR exposure induced the nuclear accumulation of RanBP9, which was prevented by ATM inhibition using KU-55933. These data indicate that RanBP9 accumulates into the nucleus in response to DNA damage, and that this accumulation is dependent on the ATM kinase activity.

### Knockdown of RanBP9 affects DDR activation

**Figure 3:**
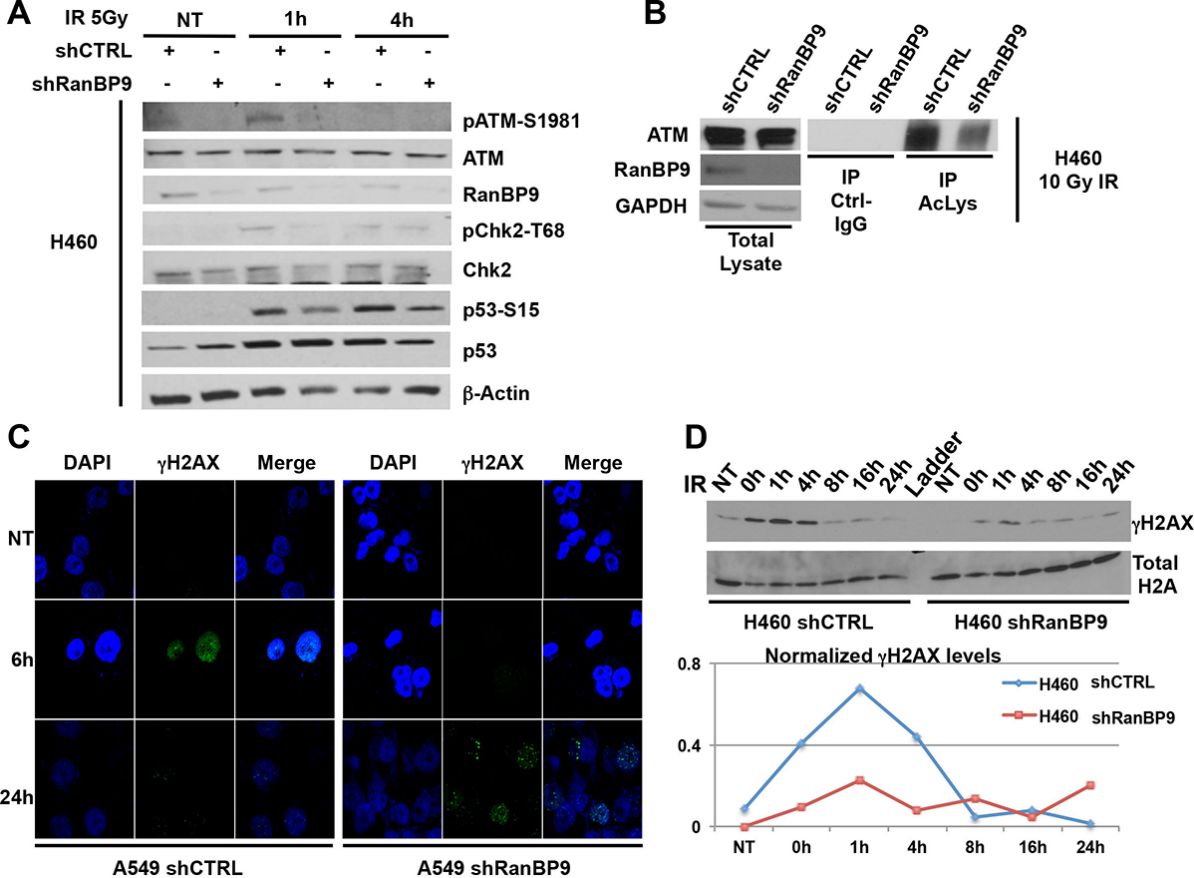
RanBP9 silencing affects the DNA-damage response (**A**) H460 cells, stably expressing control or anti-RanBP9 shRNA, were treated as indicated, harvested at different time points and analyzed by WB using the indicated antibodies. β-Actin was used as loading control. (**B**) Following 10 Gy of IR exposure, total cell extracts from H460 cells, stably expressing control or anti-RanBP9 shRNA, were immunoprecipitated using anti-pan-Acetyl-Lysine (Ac-Lys) antibody and analyzed by WB using the indicated antibodies. GAPDH was used as loading control for total lysates. (**C**) A549 cells, stably expressing control or anti-RanBP9 shRNA, were treated with 10 Gy of IR, then fixed at the indicated time points and stained using anti-phosphoH2AX (γH2AX) antibody for immunofluorescence visualization. Nuclear staining (DAPI) and merged images are also shown. (**D**) (Upper panel) H460 cells, stably expressing control or anti-RanBP9 shRNA, were treated or not with 10 Gy of IR and harvested at different time points (0–24 h). Histone proteins were extracted and analyzed by WB with a specific anti-γH2AX antibody. Total H2A was used as loading control. (Lower panel) Quantification of H2AX phosphorylation levels (γH2AX) at the indicated time-points following IR exposure analyzed by western blot using histone extracts from the upper panel.

ATM is the pinnacle kinase in the activation of the DDR following DNA DSBs [8]. To evaluate the potential role of RanBP9 in the regulation of ATM-dependent activation of the DDR, we generated stable clones from three lung cancer cell lines expressing a negative control shRNA or a RanBP9-silencing shRNA. WB and Real Time PCR analysis (Supplementary Figure 2A and 2B, respectively) confirmed strong down-regulation of RanBP9 expression in all the selected clones. Stable shRanBP9 clones were exposed to IR to activate the DDR, and harvested at different time points (1–4 h) to evaluate the phosphorylation status of ATM itself (S1981) and two of its targets (Chk2, on T68; p53 on S15) [8, 15]. As shown in Figure 3A and Supplementary Figure 2C–2D, after 1 h from IR exposure control clones showed significant Chk2, p53, and ATM phosphorylation. On the contrary, stable silencing of RanBP9 decreased their phosphorylation levels in H460 and A549 cells. When RanBP9 was silenced, we also observed reduced phosphorylation of ATM and Chk2 in response to IR in H1299 cells, a known p53-null cell line [48]. Interestingly, at 4 h from IR, differences in Chk2, p53 and ATM phosphorylation were less evident, suggesting that RanBP9 might be involved in the initial steps of DDR, modulating its efficiency and timing of activation. Recently, RanBP9 has been identified as a binding partner of KAT5 [49], a lysine acetyltransferase that binds to trimethylated histone H3 lysine 9 (H3K9me3). KAT5 activates ATM through its acetylation following IR exposure [14, 17]. Therefore, we evaluated whether RanBP9 might play a role in modulating ATM acetylation. As shown in Figure 3B, reduced ATM acetylation was observed in RanBP9 stably silenced H460 cells compared to control clones. These observations indicate that acetylation and full activation of ATM are affected by the absence of RanBP9. ATM also plays a key role in chromatin remodeling, which provides multi-protein complexes with access to DSB sites for DNA repair [3, 8, 15]. Phosphorylation of H2AX histone proteins by ATM, leading to the formation of the so-called γH2AX foci, represents a molecular marker of active DDR and is a crucial step in chromatin remodeling for efficient DNA-damage repair. We evaluated γH2AX foci by immunofluorescence in A549 clones where RanBP9 was stably silenced. As Figure 3C shows, 6 hours after IR exposure control clones display formation of clear γH2AX foci. However, the formation of γH2AX foci was strongly reduced in cells silenced for RanBP9. Conversely, at longer time points (24 h), γH2AX foci were largely absent in control clones, but were still present in RanBP9-silenced clones. To further confirm these observations, stably transfected shRanBP9 or control shRNA H460 cells were exposed to 10 Gy of IR, harvested at different time points and histone proteins were extracted. As shown by WB, γH2AX levels in cells lacking RanBP9 were significantly reduced at early time points (1–4 h). However, at 24 h, higher levels of γH2AX were observed in RanBP9 stably silenced H460 cells in comparison with control clones (Figure 3D). These results, in line with those shown in Figure 3C, demonstrate that the marked down-modulation of RanBP9 delays and reduces the activation of the DNA-repair machinery, resulting in persistence of damaged DNA for longer times. Importantly, these results are also in agreement with a recent report showing that RanBP9-deficient germ cells have increased levels of γH2AX staining [39].

### Knockdown of RanBP9 decreases homologous recombination-directed DNA repair

**Figure 4:**
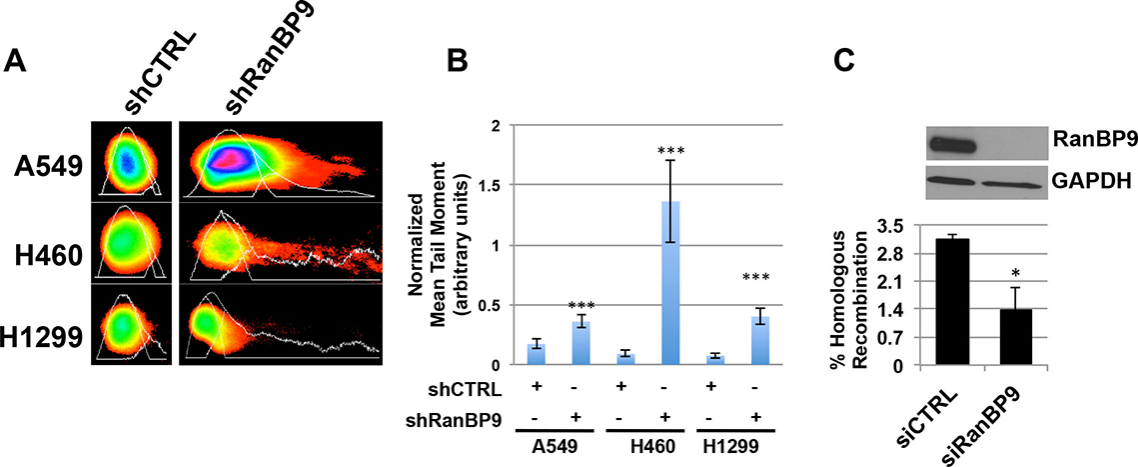
RanBP9 silencing affects DNA-damage repair mechanisms (**A**) Representative images of comets, obtained from indicated stable clones 2 h following exposure to 5 Gy of IR, analyzed using the CometScore software. (**B**) Quantitative analysis of comets shown in (A). Mean tail moment (normalized for mean tail moment evaluated immediately following IR) is reported. In each experiment, at least 50 comets were measured for each experimental point. The mean tail moment + SEM, normalized for the values obtained from untreated cells, is reported. Data are representative of two independent experiments. ***p < 0.001. (**C**) Homologous recombination assay using HeLa-DR-13-9 transfected with control (siCTRL) or anti-RanBP9 siRNA (siRanBP9). Two inactive GFP alleles were integrated in a single locus in the genome of HeLa cells (HeLa-DR13-9). Since one of the alleles contains the recognition sequence for the *I-SceI* endonuclease, transfection of a plasmid expressing this restriction enzyme results in double-strand break of one GFP allele, which can be repaired by HR using the second inactive allele of GFP. If HR-mediated DNA-repair occurs, the recombination generates an active GFP allele that can be revealed by detection and quantification of green-fluorescent cells. HeLa-DR13-9 were transfected with either a control or anti-RanBP9 siRNA and, two days later, with the plasmid encoding for the *I-SceI* endonuclease to induce DNA damage. After generating a double-strand DNA break by expressing the *I-SceI* endonuclease, functional homologous recombination results in the conversion of cells to positive for GFP. The percentage of GFP-positive cells was determined by flow cytometry (lower panel). WB analysis was performed to evaluate the down-regulation of RanBP9 expression, and GAPDH was used as loading control (upper panel). For each experimental point, at least 10,000 cells were analyzed. Data are representative of two independent experiments performed in triplicate and mean + SEM is reported. *p < 0.05.

Our data so far indicated that a strong reduction of RanBP9 affects the activation of ATM-dependent DDR signaling cascade and suggested a potential alteration of DNA repair itself. We tested this hypothesis using our cell lines where RanBP9 expression is stably silenced. Clones were exposed to IR, allowed to recover for 2 h, and then analyzed by Comet assay. As Figure 4A–4B shows, strong reduction of RanBP9 significantly impaired the ability of cells to efficiently repair DNA damaged by IR.

Since ATM plays a well-characterized role in HR-mediated DNA-repair, we evaluated the effect of RanBP9 silencing on this specific repair mechanism. To this end, we took advantage of the established *I-SceI*-GFP assay previously described [50], using HeLa-DR13-9 cells transiently transfected with anti-RanBP9 siRNAs. The percentage of GFP-positive cells (reflecting the efficiency of HR-mediated DNA-repair) was then assessed by flow cytometry. As shown in Figure 4C, silencing of RanBP9 (evaluated by WB, Figure 4C, upper panel) resulted in about 50% reduction of GFP-positive cells (Figure 4C, lower panel).

Taken together, these results indicate that absence of RanBP9 negatively affects DNA-repair at least in part by reducing effective homologous recombination.

### Knockdown of RanBP9 enhances IR-induced cellular senescence

**Figure 5:**
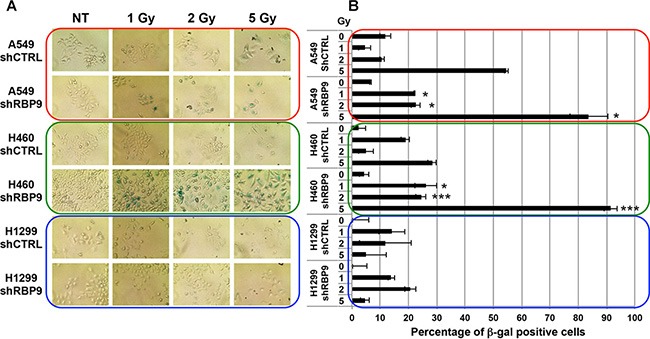
RanBP9 silencing affects IR-induced cellular senescence (**A**) Senescence-associated β-Galactosidase staining of indicated stable clones at 72 h following IR treatment. (**B**) Quantitative analysis of the experiment described in (A). Average percentage + SEM of senescence-associated β-Galactosidase positive cells for each condition is reported. Al least 20 fields for each experimental point were analyzed. *p-value < 0.05; **p-value < 0.01; ***p-value < 0.001 respect to shCTRL transfected cells, treated with the same IR dosage.

Several reports indicate that persistence of DDR activation leads to premature cellular senescence [51]. Therefore, we evaluated the effects of stable RanBP9-silencing on radiation-induced senescence in A549, H460 and H1299 cells. Stable clones were exposed to increasing amounts (1, 2 and 5 Gy) of IR and stained at 72 h for senescence-associated β-galactosidase (SA-β-gal), an established marker of cellular senescence. As shown in Figure 5, strong reduction of RanBP9 did not affect the activation of SA-β-gal in untreated (NT) cells. However, IR exposure induced cellular senescence, as indicated by SA-β-gal positive cells, in both A549 and H460 cells, but not in H1299 cells. Interestingly, silencing of RanBP9 resulted in a significant increase of SA-β-gal positive cells following IR exposure in both RanBP9 stably-silenced A549 and H460 cells, compared to their negative controls, respectively (Figure 5A–5B). These data suggest that the absence of RanBP9 cause an abnormal activation of genotoxic stress-induced senescence.

### RanBP9 is involved in cancer cell survival following genotoxic stress

**Figure 6:**
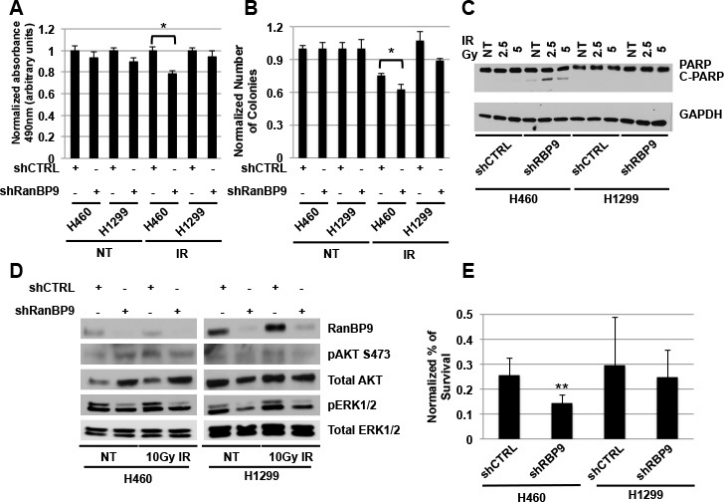
RanBP9 silencing affects cell growth and survival following genotoxic stress exposure (**A**) MTS analysis on A549, H460 ad H1299 clones, stably expressing a negative control or an anti-RanBP9 shRNA, left untreated or exposed to 5 Gy of IR at 72 h following treatment. Average of the absorbance measured at 490 nm + SEM normalized for shCTRL transfected cells, is reported. *p-value < 0.05; Reported data are representative of at least two independent experiments performed in triplicate. (**B**) Quantitation of colony assay using stable clones as in A, left untreated (NT) or exposed to 1 Gy of IR. Cells were grown for 10 days and colonies were stained using crystal violet and counted. Average colony number + SEM, normalized for the untreated control, is reported. *p-value < 0.05; data are representative of two independent experiments performed in triplicate. (**C**) WB analysis of stable clones as in (A) left untreated (NT) or exposed to 2.5 or 5 Gy of IR and harvested at 24 h following treatment. Total cell extracts were analyzed using anti-PARP antibody raised against total and cleaved (active) PARP (C-PARP). GAPDH was used as loading control. (**D**) WB analysis of stable clones as in (A–B) left untreated (NT) or exposed to 10 Gy of IR and harvested at 4 h following treatment. Total cell extracts were analyzed using the indicated antibodies. Total ERK1/2 was used as loading control. (**E**) Indicated stable clones were treated with 20 μM cisplatin for 48 h and cell sensitivity to the treatment was assessed by direct cell counting. Percentage of cell survival was normalized for relative untreated controls. **p-value < 0.01 compared to the shCTRL transfected clone; data are the average of at least two independent experiments performed in triplicate.

The prompt activation of DDR by ATM following exposure to IR is crucial for cancer cells to engage in DNA repair and prevent the activation of programmed cell death [3, 8]. The effect of RanBP9 silencing on ATM-dependent pathways led us to hypothesize that a reduction of RanBP9 may affect also proliferation and survival of cells exposed to IR. To test this hypothesis, we performed different cell proliferation and survival assays, including MTS (Figure 6A), colony assay (Figure 6B) and cell count analysis (Supplementary Figure 3) using H460 and H1299 stable clones described above, exposed to IR. In accordance with our previous results, knockdown of RanBP9 decreased cell proliferation, colony formation, and survival of shRanBP9 transfected H460 cells compared to control transfected cells exposed to IR. Furthermore, evaluation of total and active PARP (C-PARP), one of the markers of terminal stages of apoptosis activation, revealed that following IR treatment, RanBP9-silenced H460 cells displayed higher levels of C-PARP compared to the control cells (Figure 6C). Of note, we did not observe the same reduction of cell proliferation, survival, or the increased apoptosis in RanBP9-silenced H1299 cells compared to control cells (Figure 6A–6C, Supplementary Figure 3).

We also evaluated whether AKT1 or ERK1/2 phosphorylation, two widely-used markers of apoptosis inhibition and activation of proliferation, might explain the differential response of RanBP9-silenced cells to IR. As shown in Figure 6D, RanBP9 abrogation by shRNA stable transfection resulted in reduced ERK1/2 activation in both cell lines. This result is in line with the previously described role of RanBP9 in the activation of the Ras/ERK pathway [37]. On the other hand, we found that AKT phosphorylation did not show any difference in silenced cells compared to controls, although the total levels of AKT were increased in RanBP9-silenced H460 cells but not in RanBP9-silenced H1299 cells compared to controls.

Finally, we tested whether RanBP9 expression affects the sensitivity to other genotoxic treatments, currently used in the clinic for lung cancer therapy, such as cisplatin. Figure 6E shows that stable knockdown of RanBP9 results in enhanced sensitivity to cisplatin, compared to control shRNA-transfected cells, in a cellular context-dependent manner, in agreement with other observations obtained following IR treatments (Figure 6A–6B). Taken together these data indicate that the reduced activation of DDR observed when RanBP9 is silenced results in an enhanced sensitivity of lung cancer cells to different genotoxic stress in a cellular context-dependent manner, and that different pathways, including, at least in part, ERK1/2 and AKT, might be involved in the RanBP9-dependent regulation of cellular DDR, proliferation and apoptosis.

## DISCUSSION

A better understanding of the molecular mechanisms of the cellular response to DNA-damaging agents is required to increase the efficacy of current anti-neoplastic treatments [22, 23, 52]. In the present study, we investigated the role of RanBP9 in the response and sensitivity to DNA damage of lung cancer cells exposed to IR. RanBP9 is a ubiquitously expressed protein that has been previously linked to exposure to genotoxic stress [43, 44]; however, its biological functions in normal and cancer cells are still unknown [30–34]. The present study lays the foundations for future investigations to evaluate in more depth the role of RanBP9 as a player in the DDR and as a potential marker of cellular sensitivity to genotoxic treatments in appropriate cellular contexts.

An initial analysis of the amino acid sequence revealed the presence on RanBP9 of several putative phosphorylation sites for major kinases involved in cellular DDR (Figure 1A–1B, Supplementary Table 1). This finding is in agreement with a large-scale screening of ATM substrates, reporting RanBP9 S603 as a potential target of ATM kinase activity [45]. Here, we show a significant interaction between active ATM and RanBP9 in lung cancer cells at early time points following IR exposure (Figure 1D, Supplementary Figure 1B). We also found that, *in vitro*, ATM phosphorylates at least two (S181 and S603) sites on RanBP9 (Figure 1C). However, we cannot exclude the possibility that ATM might also phosphorylate additional RanBP9 residues. Further, since we also used ATM immunopurified from cell extracts (Figure 1C–1D), we cannot rule out the possibility that other proteins including kinases involved in the cellular DDR co-immunoprecipitated and contributed to the phosphorylation of RanBP9.

RanBP9 is found in both the nucleus and the cytoplasm and a recent report identified different regions of the protein that might be involved in its nuclear localization [34]. However, the molecular mechanisms regulating RanBP9 subcellular localization are not understood. Here, we demonstrate that, following IR exposure, RanBP9 promptly (1–8 h) localizes into the nucleus (Figure 2). This nuclear accumulation is no longer present at later time points (48 h) and is prevented when the ATM kinase is specifically inactivated (Figure 2D). The latter observation suggests that ATM phosphorylation might either signal the nuclear accumulation of RanBP9 or block its cytoplasmic re-localization. Since the analyzed cellular systems displayed slightly different timing of RanBP9 nuclear accumulation, it can be hypothesized that cell type specific factors other than ATM might be involved in the regulation of RanBP9 nuclear/cytoplasmic delocalization in response to DNA damage. Future studies will be required to investigate whether ATM phosphorylation affects RanBP9 subcellular localization by modifying its tridimensional structure and/or altering its interaction with partners involved in nucleus-cytoplasm shuttling. Notably, *Schild-Poulter* and her group recently reported that RanBP9 localizes to the cytoplasm 24–72 h after exposing cells to IR [30, 33]. Our findings complement those observations and suggest that, in the early stages of the DDR, RanBP9 undergoes an ATM-mediated nuclear enrichment, followed by a latter cytoplasmic re-localization.

Based on our initial findings, we speculated that ATM-dependent nuclear localization of RanBP9 might contribute to activate DDR and DNA repair. To explore this hypothesis, we generated stable clones of lung cancer cell lines of different origins in which RanBP9 expression was stably silenced. Following IR exposure, ATM-pS1981 levels and phosphorylation of ATM downstream targets, including Chk2, p53 and H2AX, were reduced in the absence of RanBP9 (Figure 3A, Supplementary Figure 2C–2D). Importantly, RanBP9 knockdown decreased levels of acetylated ATM (Figure 3B). These observations suggest that the efficiency of the DDR is compromised by the absence of RanBP9. In fact, time-course experiments indicated that, in RanBP9-silenced cells, ATM-dependent phosphorylation of H2AX, a marker of active DNA damage response, was significantly decreased at shorter time points, but lasted longer (Figure 3C–3D), compared to control cells. Importantly, these results are in accordance with the observation that RanBP9-deficient germ cells show increased levels of γH2AX staining [39]. Intriguingly, this phenotype is also similar to the delay in disappearance of γH2AX foci when KAT5 is silenced (see below) [53].

Following DNA-damage, ATM activates a signaling cascade that leads to the immediate activation of DNA-repair mechanisms, mostly through Homology-Directed DNA repair [3, 8]. Our data show that knockdown of RanBP9 negatively affected the ability of lung cancer cells to repair damaged DNA by HR (Figure 4). When RanBP9 expression was abrogated, defective DDR resulted in decreased cellular proliferation, activation of senescence or apoptosis following IR treatment (Figures 5–6, Supplementary Figure 3). Similar results were also observed following cellular exposure to other types of genotoxic stress such as cisplatin treatment. These effects, however, were not observed in H1299 cells, indicating that other factors, downstream of RanBP9, are involved in these cellular pathways. One possible explanation is that this cellular system lacks p53, which plays a key role as mediator of cellular senescence and apoptosis following genotoxic stress [54]. The observation that RanBP9 abrogation enhances the sensitivity to genotoxic treatments (IR and cisplatin) of specific lung cancer cells invites to speculate that compounds inhibiting RanBP9 activity in DDR might represent a new strategy to induce synthetic lethality. These theoretical compounds could be used in association with other drugs (i.e. PARP inhibitors) targeting the cellular DNA repair machinery. However, since the role of RanBP9 in cellular proliferation, apoptosis and DDR might be dependent on the cellular context, further studies are required to shed more light on the molecular mechanisms affected by RanBP9 abrogation and its direct and/or indirect molecular partners.

All together, our results showed that RanBP9 is critically involved in the early steps of DDR in lung cancer cells, acting both as target and co-regulator of ATM in the effective activation of the DNA repair machinery. Interestingly, other proteins, (e.g. c-Abl), have similar interactions with ATM [55, 56]. Recently, *Domingues et al.* reported that RanBP9 is a binding partner of the lysine-acetyltransferase KAT5/Tip60, a major player of the DDR [49]. Following IR exposure, KAT5 acetylates ATM, promoting its activation and the DDR [14, 17]. Although it is established that KAT5 acetylates ATM, there is no proof of their direct interaction [57]. Indeed, the existence of unidentified mediator(s) required to modulate KAT5 acetylation of ATM is hypothesized [58]. Our results indicate that the absence of RanBP9 affected the acetylation levels of ATM following IR exposure. Therefore, it can be speculated that RanBP9 is involved in KAT5-mediated activation of ATM, acting as a scaffold that bridges the two proteins together. Establishing what is the exact molecular mechanism through which RanBP9 facilitates ATM full activation will require future investigations.

In summary, our study identifies RanBP9 as a new target of ATM, critically involved in the prompt activation of DDR signaling, repair of damaged DNA and cancer cell sensitivity to genotoxic stress.

## MATERIALS AND METHODS

### Cell cultures, transfections, plasmids, and treatments

A549, H460 and H1299 cells were purchased from ATCC and cultured in RPMI-1640 medium supplemented with 10% FBS and penicillin/streptomycin. For stable clones generation, cells were transfected using SureSilencing shRNA plasmids (Qiagen, Valencia, CA, USA) and cells stably integrating the silencing plasmid were selected and sub-cultured in medium containing 1 mg/ml of puromycin. HeLa-DR13-9 cells were cultured as previously described [50]. Plasmid and siRNA transfections were performed using Lipofectamine-2000 or Oligofectamine (Invitrogen, Carlsbad, CA, USA), respectively, according to manufacturer's instructions. Control and anti-RanBP9 siRNAs were purchased from Santa-Cruz (Santa Cruz, CA, USA). Plasmid for transient expression of RanBP9-GFP fusion protein, cloned in pCMV6-AC-GFP vector, was purchased from Origene. For irradiation experiments, cells were treated using an X-ray linear accelerator (Gammacell 40 Exactor, Best Theratonics, Ottawa, Canada) with doses ranging from 1 to 10 Gy. For ATM inhibition, cells were treated with 10 μM KU-55933 (EMD-Millipore, Billerica, MA, USA) for 1 h before ATM activation.

### Protein extractions, nuclear/cytoplasmic fractionations, immunoprecipitations, and western blots

Total protein extractions were performed as previously described [59] Nuclear/cytoplasmic protein fractionations were performed using the NE-PER Nuclear and Cytoplasmic Extraction Reagents (Thermo, Waltham, MA, USA), according to manufacturer's instructions. Histone extraction [60] and immunopreciptiation [59] experiments were performed as previously described. Western blot analyses were performed according to standard protocols. Antibodies used were anti-ATM S1981p (Rockland, Philadelphia, PA, USA), anti-p53 DO-1, anti-p-p53 (Ser15), anti-ATM 2C1 (Santa Cruz), anti-RanBP9 (Abcam, Cambridge, MA, USA), anti-pChk2 (Thr68), anti-Chk2, anti-γH2AX, anti-H2A, anti-PARP, anti-GAPDH-HRP-conjugated, anti-β-actin (Cell Signaling Technology, Danvers, MA, USA) and anti-p84 (GeneTex, San Antonio, TX, USA). ImageQuant software (Biorad, Hercules, CA, USA) was used for the quantification of western blot data.

### Kinase assay

Radioactive kinase assay was conducted as previously described [61]. Briefly, 20 ng of recombinant active ATM kinase (purchased from EMD Millipore, Billerica, MA) was resuspended in ATM kinase buffer (20 mM HEPES (pH 7.5), 50 mM NaCl, 10 mM MgCl_2_ and 10 mM MnCl_2_). ATM kinase reactions were carried out at 30°C for 15 min in 50 μl of kinase buffer containing 20 μCi of [γ-^32^P] ATP and 200 ng of *in vitro* synthesized peptides indicated in Figure 1B (Thermo). Following reaction, peptides were spotted onto P81 phosphocellulose squares (EMD Millipore) and washed 4 times with 75 mM orthophosphoric acid. ^32^P incorporation was measured by a β-counter scintillator.

Non-radioactive kinase assay experiments were performed using the Kinase Glo Luminescent Kinase assay kit (Promega, Madison, WI, USA) as suggested by the manufacturer. ATM kinase was purified from H460 lung cancer cells untreated or irradiated with 10 Gy of IR in the presence or in the absence of KU-55933, as previously reported [13]. One tenth of the volume was analyzed by western blot. Peptides indicated in Figure 1, or their mutant where putative target sites were mutated into alanine (*in vitro* synthesized by Thermo), were added to the kinase reaction (1 mg per reaction) along with 1 μM ATP, and incubated at 32°C for 15 minutes. To block kinase activity, Kinase Glo reagent was added to the reaction. The reagent also contains recombinant luciferase and luciferin. Luciferase converts the luciferin into oxyluciferin in an ATP-dependent manner, and generates a luminescent signal that depends on the amount of ATP not used by the immunopurified kinase in the kinase reaction. Lower luminescent signal represent lower levels of ATP left in solution, and in turn higher kinase activity. Luminescent signal was detected using a GloMax 96 microplate luminometer (Promega). Luminescent signal obtained from immunopurified ATM was normalized for the luminescent signal obtained from control IgG-precipitated lysates and for the signal obtained from the mutant peptides.

### Immunofluorescence

Immunofluorescence staining of γH2AX was performed as previously described [62], and analyzed using an FV1000 Confocal Microscope (Olympus, Center Valley, PA, USA).

### Live-imaging

For RanBP9 localization experiments using live-imaging microscopy, H460 cells were transfected as described above with GFP (not shown) or RanBP9-GFP expression vectors. At 24 h from transfection, cells were left untreated (not shown) or irradiated with 10 Gy of IR and analyzed using an Eclipse Ti-E microscope (Nikon). At least 5 different fields were acquired every 5 minutes for 10 hours. Images were processed using the NIS-Elements Viewer software (Nikon), and green fluorescence was converted in orange pseudo-color to visualize the accumulation of GFP proteins. Fluorescence was also quantitated using the same software.

### Quantitative real time PCR (qRT-PCR)

qRT-PCR experiments were performed using the TaqMan Fast-PCR kit (Applied Biosystems, Waltham, MA, USA) according to the manufacturer's instructions, using the appropriate TaqMan probes for mRNA quantification. All reactions were performed in triplicate. Simultaneous quantification of GAPDH mRNAs was used as reference for mRNA quantification. The Ct-method for relative quantification of gene expression (User Bulletin #2; Applied Biosystems) was used to determine mRNA expression levels.

### Comet assay

Indicated stable clones cell lines were irradiated with 5 Gy of IR or left untreated, and were allowed to repair the DNA for 2 h in complete medium. Cells were then harvested and processed using the alkaline COMET assay (Trevigen, Gaithersburg, MD, USA) according to manufacturer's instructions. Comet images were analyzed using COMET Score (TriTrek, Annandale, VA, USA). Comet tail moment was used as the measure of DNA damage.

### Homologous recombination assay

HR assays were performed as previously described [50]. Briefly, on day 1 HeLA-DR13-9 cells were transfected with 30 pmol of control or anti-RanBP9 siRNA. On day 2, cells were transferred to 35 mm dishes. On day 3, cells were transfected with 50 pmol of control or anti-RanBP9 siRNA along with *I-SceI* expression vector to induce DNA DSB. On day 6, cells were harvested and GFP positive cells were counted using a FACS Calibur flow cytometer (Becton-Dickinson).

### Senescence assay

Indicated sub-confluent stable clones were left untreated or irradiated using the indicated amounts of IR. At 72 h following IR, Senescence Associated β-galactosidase (SA-β-gal) activity was assessed by using the Senescent Cells Staining kit (Cell Signaling) following the manufacturer's instructions. Cells were analyzed with light microscopy to determine percentage of SA-β-gal-positive senescent cells.

### Cell survival assays

For MTS assays, 1 × 10^4^ cells (for the indicated cell lines) were plated in 96-well plates and treated with 5 Gy of IR and assays were performed at 72 h following the treatment, using the CellTiter96 Aqueous One Solution kit (Promega), according to the manufacturer's instructions. Absorbance at 490 nm was evaluated using a SpectraMax M5 microplate reader (Molecular Devices, Sunnyvale, CA, USA).

For viability assays, 1 × 10^5^ cells (for the indicated cell lines) were plated in 12-well plates and treated with the indicated amounts of IR. At 72 h following irradiation, cells were harvested, and counted using a hemocytometer.

For colony assay experiments, 300 cells (for the indicated cell lines) were plated in 6-well plates and treated with 1 Gy of IR or left untreated. Cells were grown for 10 additional days, to allow the formation of the colonies and stained with crystal violet. Colonies were counted, and data, normalized for the non-treated samples + SEM, were reported.

### Statistical analysis

All the experiments are representative of at least two independent experiments. Student's *t* test was used to determine the statistical significance (indicated as *p*-value) for each quantitative experiment. All error bars represent the standard error of the mean (SEM). Data were considered statistically significant for *p* < 0.05, at least.

## SUPPLEMENTARY FIGURES, VIDEO AND TABLE




