# Supplementary material for: Ran Binding Protein 9 (RanBP9) is a novel mediator of cellular DNA damage response in lung cancer cells
Source: Oncotarget. 2016 Mar 1;7(14):18371–83. doi: 10.18632/oncotarget.7813 (PMC4951294; doi:10.18632/oncotarget.7813)
Supplement: Supplementary file 1 [file oncotarget-07-18371-s001.pdf]

Ran Binding Protein 9 (RanBP9) is a novel mediator of cellular DNA damage response in lung cancer cells

Supplementary Materials

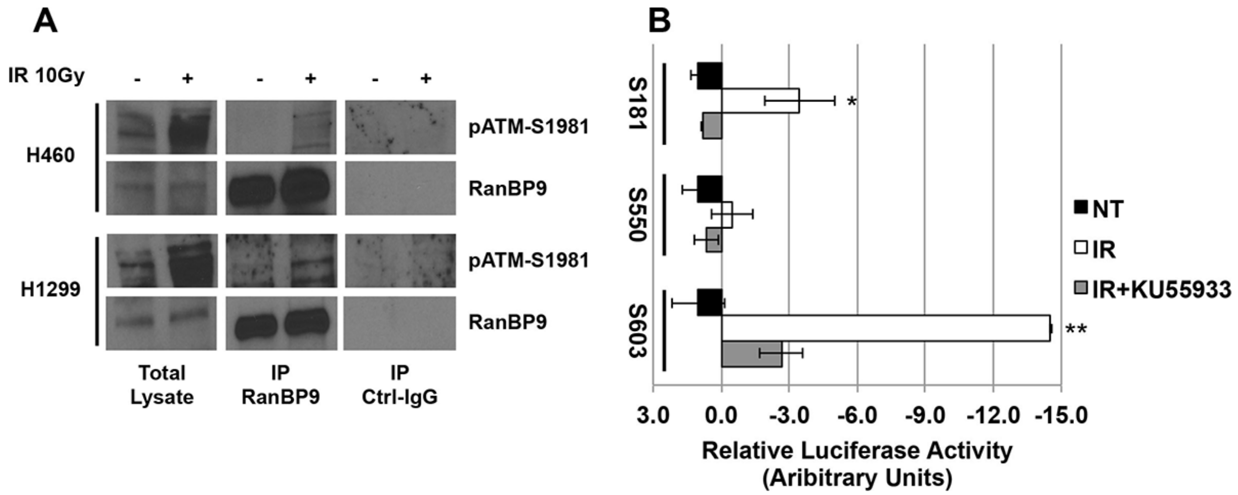

Supplementary Figure S1: RanBP9 is a novel target of ATM. (**A**) Co-immunoprecipitation experiments in H460 and H1299 lung cancer cells. Cells were irradiated as indicated, harvested after 10 minutes and total cell extracts were immunoprecipitated (IP) using anti-RanBP9 or normal control (Ctrl) IgG. Total and immunoprecipitated cell extracts were analyzed through WB using the indicated antibodies. (**B**) Kinase assay using immunopurified ATM from H460 cells treated with 10 Gy of IR or left untreated. Where indicated, KU-55933 was used to inhibit ATM. Immunopurified ATM was incubated for 15′ at 32°C with the indicated peptides or their mutant forms where each putative phosphorylated serine was substituted with alanine (reported in B) in the presence of ATP. Following the kinase reaction, luciferin and luciferase were added to generate a luminescent signal depending on the amount of ATP left in the solution at the end of the reaction. Lower levels of luciferase activity correspond to lower levels of ATP left in solution and to a higher kinase activity. Data were normalized for both luciferase activity obtained when lysates where immunoprecipitated using normal control IgG and when S/A mutants where used. Luciferase activity, relative to the untreated samples, is reported. Data are representative of 2 independent experiments performed in triplicate, ± SEM. * $p<0.05$; ** $p < 0.01$.

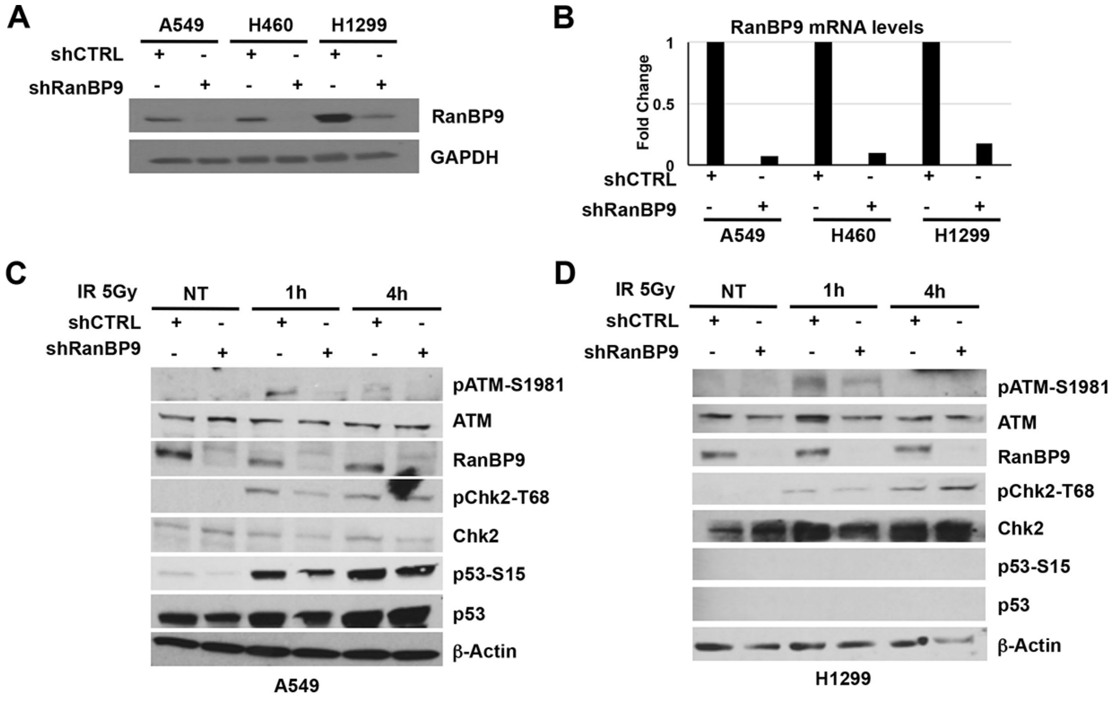

Supplementary Figure S2: RanBP9 silencing affects the DNA-damage response. (**A**–**B**) WB (A) and Real-Time (B) analysis of A549, H460 ad H1299 clones stably expressing a Negative control or an anti-RanBP9 ShRNA. For western blot, GAPDH was used to normalize total protein levels. For Real-Time analysis, RanBP9 expression levels were normalized using GAPDH gene expression levels as a control. (**C**–**D**) H460 (C) ad H1299 (D) clones stably expressing a Negative control or an anti-RanBP9 ShRNA were treated as indicated and harvested at different time points and analyzed by western blot using the indicated antibodies. GAPDH was used as loading control.

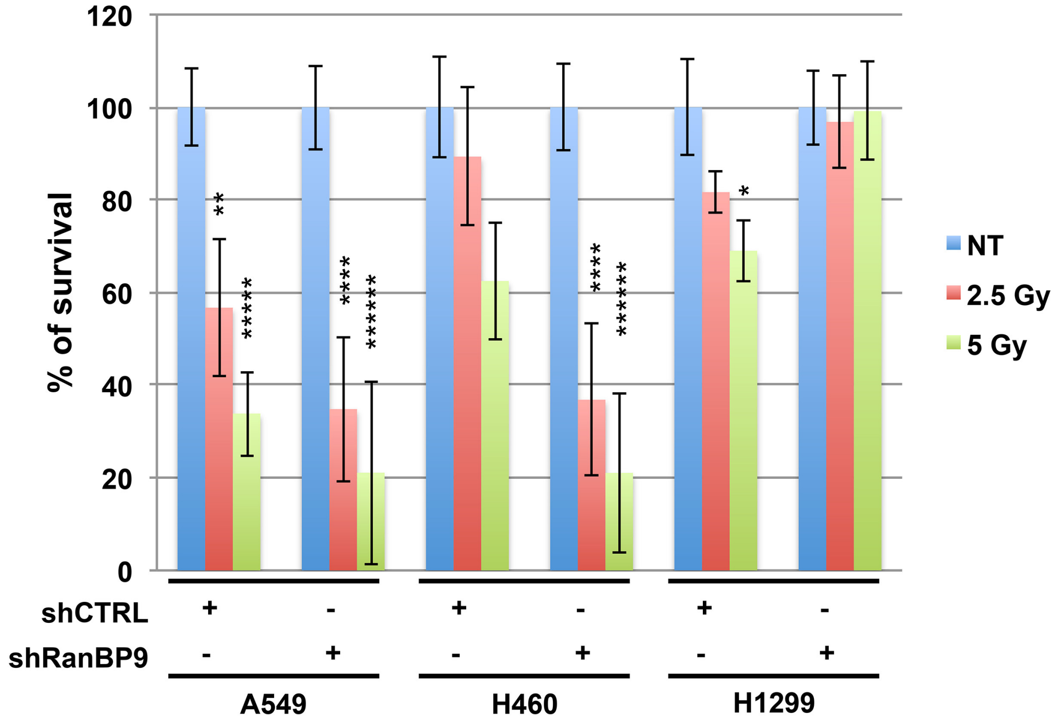

Supplementary Figure S3: RanBP9 silencing affects cell growth following IR exposure. Cell growth assay on indicated stable clones left untreated (NT) or exposed to 2.5 or 5 Gy of IR at 72 h following the treatment. Average cell number ± SEM, normalized for the untreated control, is reported. **p*-value < 0.05, ***p*-value < 0.01; *****p*-value < 0.0001; ******p* < 0.00001; ******p*-value < 0.000001; *******p*-value < 0.0000001. Data are the average of two independent experiments performed at least in triplicate.

Supplementary Video S1: RanBP9 accumulates into the nucleus following IR exposure. H460 cells were transfected using a RanBP9-GFP expression vector. At 24 h from the transfection, cells were exposed to 10 Gy of IR and analyzed by live-imaging confocal microscopy for 6 h. Images were acquired every 5 minutes and the resulting video was processed using Nikon Element Viewer Software.

Supplementary Table S1: List of potential sites on RanBP9 predicted to be phosphorylated by major kinases involved in DDR

Residue		Kinase	Sequence	Score
46	S	CAMK/RAD53/CHK2	PAVSAGSSPAGSPGG	3
128	S	CAMK/RAD53/CHK2	PALVAGSSAAAPFPH	2.941
181	S	Atypical/PIKK/ATM	KFSYIGLSQNNLRVH	4.825
300	S	CAMK/CAMKL/CHK1	FYTKNGHSLGIAFTD	3.357
374	S	CAMK/RAD53/CHK2	TMIQKMVSSYLVHHG	2.765
374	S	CAMK/CAMKL/CHK1	TMIQKMVSSYLVHHG	4.143
426	T	Atypical/PIKK/DNAPK	MGEAIETTQQLYPSL	2.857
470	S	Atypical/PIKK/ATR	LGGRSPKSQDSYPVS	7.9
470	S	Atypical/PIKK/DNAPK	LGGRSPKSQDSYPVS	3.524
477	S	Atypical/PIKK/DNAPK	SQDSYPVSPRPFSSP	2.095
483	S	Atypical/PIKK/DNAPK	VSPRPFSSPSMSPSH	2.238
485	S	CAMK/RAD53/CHK2	PRPFSSPSMSPSHGM	2.882
534	S	CAMK/CAMKL/CHK1	CHSNKHQSSNLNVPE	3.5
535	S	CAMK/CAMKL/CHK1	HSNKHQSSNLNVPEL	3.929
548	S	Atypical/PIKK/DNAPK	ELNSINMSRSQQVNN	2.81
550	S	CAMK/RAD53/CHK2	NSINMSRSQQVNNFT	3
550	S	CAMK/CAMKL/CHK1	NSINMSRSQQVNNFT	3.5
550	S	Atypical/PIKK/ATM	NSINMSRSQQVNNFT	6.175
550	S	Atypical/PIKK/DNAPK	NSINMSRSQQVNNFT	3.762
558	S	Atypical/PIKK/DNAPK	QQVNNFTSNDVDMET	3.238
603	S	Atypical/PIKK/ATM	TEMEVDSSQLRRQLC	7.754
603	S	Atypical/PIKK/ATR	TEMEVDSSQLRRQLC	9.9
603	S	Atypical/PIKK/DNAPK	TEMEVDSSQLRRQLC	2.429
613	S	Atypical/PIKK/ATR	RRQLCGGSQAAIERM	8.4
613	S	Atypical/PIKK/DNAPK	RRQLCGGSQAAIERM	2.286
683	S	CAMK/RAD53/CHK2	PVCSALNSAILETHN	2.941
